# Perioperative care of neonates with critical pulmonary stenosis: Case report

**DOI:** 10.1097/MD.0000000000037203

**Published:** 2024-02-23

**Authors:** Qiong He, Min Song, Yanping Huang, Ling Wan

**Affiliations:** a Wuhan Children’s Hospital (Wuhan Maternal and Child Healthcare Hospital), Tongji Medical College, Huazhong University of Science & Technology, Wuhan, Hubei 433000, China.

**Keywords:** multidisciplinary collaboration, neonatal, perioperative care, severe pulmonary stenosis, specialized care

## Abstract

**Rationale:**

Summarizing the perioperative nursing experience in the successful treatment of 4 neonates with critical pulmonary stenosis (CPS).

**Patient concerns:**

Of the 4 patients, 3 had postnatal shortness of breath and varying degrees of cyanosis, aggravated by crying and noise, and 1 had no obvious shortness of breath and cyanosis. The preoperative auscultation of the precordial region could be heard 3-4/6 systolic murmur; echocardiography was diagnosed as CPS, combined with patent ductus arteriosus, right ventricular dysplasia, and severe tricuspid regurgitation. Four children were treated with prostaglandin 5 ng/(kg-min) to maintain a certain degree of pulmonary blood flow to improve hypoxemia, effectively preventing ductus arteriosus from closure, and the infusion was discontinued 2 hours prior to the operation. Three of the children required ventilator-assisted respiration to relieve severe hypoxia and correct acidosis before surgery.

**Diagnosis:**

Neonatal CPS was diagnosed.

**Interventions:**

Four neonates with rapidly developing conditions were admitted to the hospital, a multidisciplinary in-hospital consultation was organized immediately, and a multidisciplinary collaborative team was set up, consisting of medical doctors and nurses from the medical department, the neonatal intensive care unit, cardiovascular medicine, cardiac ultrasound room, anesthesiology department, and radiology and interventional medicine department. The multidisciplinary team evaluated the treatment modality of the children and finally decided to perform percutaneous balloon pulmonary valvuloplasty. The surgical team included specialists from the Department of Cardiovascular Medicine, Department of Interventional Radiology, Cardiac Ultrasound Unit, and Department of Anesthesiology.

**Outcomes:**

All 4 neonates were successfully operated and discharged from the hospital. Multidisciplinary follow-up interventions were carried out 1 year after discharge, and the children were in good condition.

**Lessons:**

The specialty nursing-led multidisciplinary collaboration model significantly improves the professional competence of nurses from various specialties, promotes the integration and development of multispecialty disciplines, and provides better quality services for children, which is the key to improving the success rate of percutaneous balloon pulmonary valvuloplasty in neonates.

## 1. Introduction

The congenital heart disease (CHD) is the most common congenital malformation in newborns, with an incidence of 8% to 10% of live births.^[1,2]^ Critical pulmonary stenosis (CPS), that is, severe pulmonary valve stenosis, is a CHD that seriously jeopardizes the health of newborns and small infants. In CPS, right ventricular output is severely impaired, tricuspid regurgitation is massive, right atrial pressure is elevated, and right-to-left shunting occurs at the foramen ovale or atrial septal defect. Severe pulmonary stenosis often presents as severe heart failure in the newborn and progresses rapidly, usually requiring prompt surgery. Therefore, early identification and release of stenosis is the key to saving the lives of children with this type of CHD.^[3]^ Since its first application in 1982, percutaneous balloon pulmonary valvuloplasty (PBPV) has been accumulated and developed for more than 40 years and has gradually become the preferred treatment option for CPS.^[4]^

From 2019 to 2021, 4 cases of neonates with severe pulmonary valve stenosis were successfully treated in our hospital based on the application of the multidisciplinary team (MDT) model, and after comanagement by an MDT, the postoperative recovery was smooth, and the condition of regular follow-up after discharge was good. The nursing experience is reported as follows.

## 2. Case report

### 2.1. General information

The general information of the 4 children is shown in Table 1. Three children presented with shortness of breath, cyanosis of different degrees, aggravated by crying and noise, transcutaneous oxygen saturation (SpO2) of 60% to 80% in a quiet state, with an average oxygen saturation of 64.0% ± 8.6%, which was difficult to improve with oxygen, and 1 child had no obvious shortness of breath and cyanosis. In one case, there was no obvious shortness of breath or cyanosis. A 3-4/6 systolic murmur was detected in the precordial region on auscultation before surgery, and CPS was diagnosed by echocardiography as a combination of patent ductus arteriosus, right ventricular dysplasia, and severe tricuspid regurgitation. Four children were treated with 5 ng of prostaglandin (5 ng/(kg-min)) to maintain a certain degree of blood flow in the pulmonary circulation to improve hypoxemia, effectively preventing the ductus arteriosus from closing and stopping the infusion for 2 hours before surgery. Three of them needed ventilator-assisted respiration to relieve severe hypoxia and correct acidosis before surgery. Four neonates had rapid disease development, and a multidisciplinary in-hospital consultation was organized immediately after admission and an MDT was set up, consisting of doctors and nurses from the medical department, the neonatal intensive care unit (NICU), cardiovascular internal medicine, cardiac ultrasound room, anesthesiology, and radiology and interventional radiology department. The MDT evaluated the treatment modality of the child and finally decided to perform PBPV. The surgical team included specialists from the Department of Cardiovascular Medicine, Department of Interventional Radiology, Cardiac Ultrasound Unit, and Department of Anesthesiology (Table 1).

**Table 1:** General information about the children.

Group	Sex	Pregnancy and parity	Gestation weeks	Birth mode	Birth weight (kg)	Age at surgery
Case 1	Male	Pregnancy 1 and birth 1	38	C-section	3.37	9 days
Case 2	Female	Pregnancy 2 and birth 2	39	C-section	2.50	1 day
Case 3	Male	Pregnancy 1 and birth 1	39	C-section	3.35	2 days
Case 4	Female	Pregnancy 3 and birth 3	41	Vaginal birth	3.60	0 day

### 2.2. Treatment and regression

**Figure 1.**
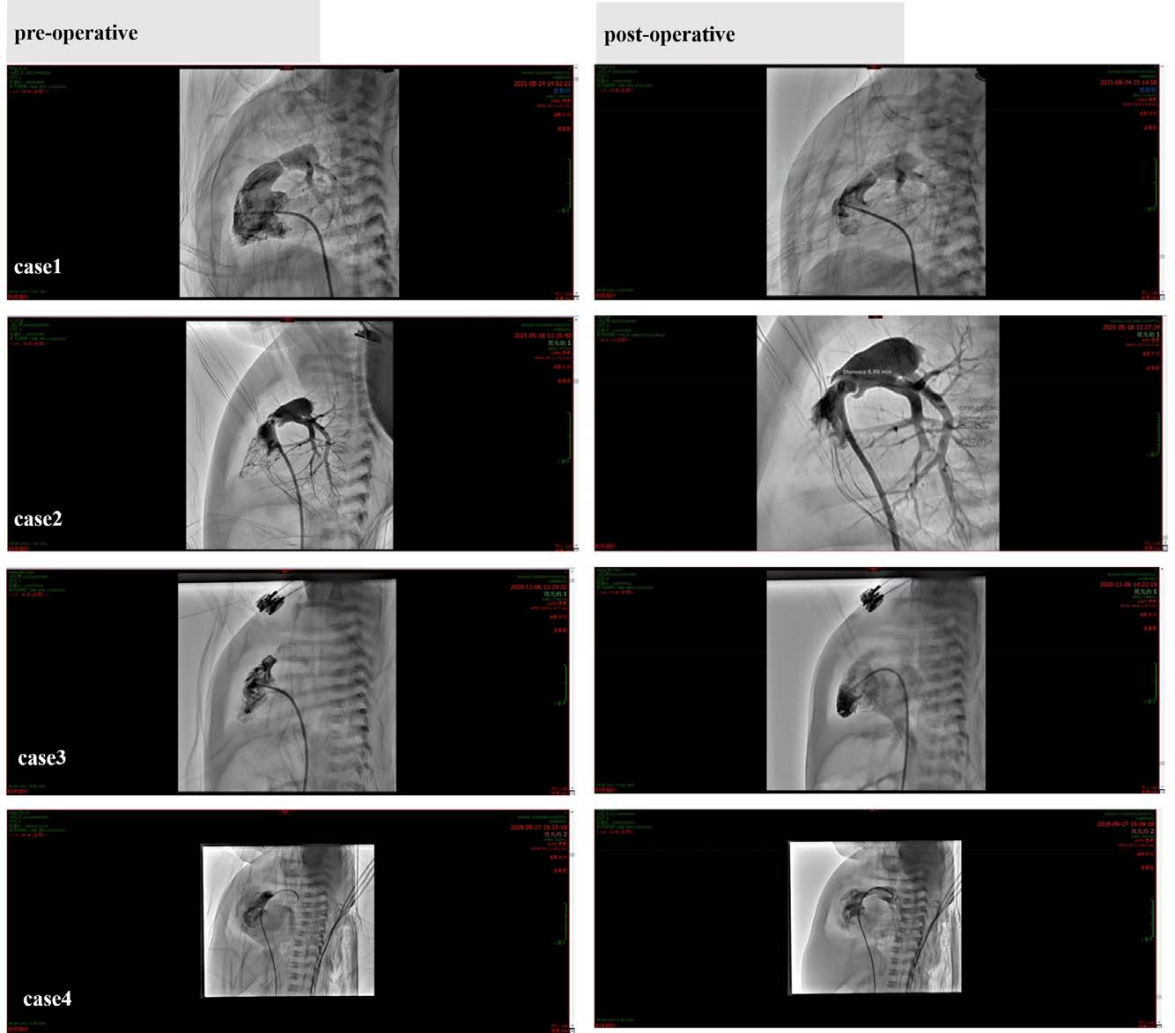
Pre- and postoperative pulmonary arteriography in 4 neonates with PBPV. PBPV = percutaneous balloon pulmonary valvuloplasty.

All 4 neonates underwent balloon dilatation and angioplasty under tracheal intubation and intravenous anesthesia and the right femoral vein was punctured, and a 6 French (F) right heart catheter was used to perform right ventriculography to clarify the position of the pulmonary valve opening and the size of the valve opening, and to measure the diameter of the pulmonary valve annulus, the systolic pressure of the right ventricle, and the pressure of the main pulmonary artery, and to assess the development of the right ventricle. The 6 F multipurpose catheter was placed in the left lower pulmonary artery or aorta along the above pathway, the balloon delivery track was established, the coronary balloon was selected to preexpand the pulmonary valve, and the balloon was seen to show a clear “waist concavity” sign, and the balloon was repeatedly filled 2 times, each time for 3 to 5 seconds. Vital signs such as heart rate, heart rhythm, SpO2, and noninvasive blood pressure were monitored during the operation. Except for one case of transient bradycardia and hypoxemia during intraoperative catheterization or ballooning into the pulmonary valve and balloon filling and dilatation, there were no serious arrhythmias, valvular injuries, cardiac tamponade, and other complications, and the surgical treatment was successful. The systolic pressure of the right ventricle decreased from 78 to 99 mm Hg (1 mm Hg = 0.133 kPa) preoperatively to 20 to 45 mm Hg postoperatively. Postoperative angiography showed a significant improvement in the opening of the pulmonary valve, and the oxygen saturation level increased from 60% to 80% preoperatively to 90% to 95% postoperatively, and the catheter was withdrawn from the sheath and the puncture site was bandaged with pressure. The children were returned to the NICU for postoperative monitoring, and perioperative cardiac monitoring was continued to closely monitor the hemodynamic situation. In this group, there was one case of postoperative hypoxemia in the early postoperative period, which recovered well after active targeted treatment, and one case of postoperative deep vein thrombosis on the side of the puncture limb, which improved after anticoagulant and symptomatic treatment. After anticoagulation and symptomatic treatment, the symptoms improved. All the test indexes were normal, and the patient was discharged from the hospital. At 1-year follow-up, cardiac ultrasound examination showed all children in good condition, only with mild regurgitation in the pulmonary valve. The following figure shows pre- and postoperative pulmonary arteriography in the 4 neonates with PBPV (Fig. 1).

## 3. Discussion

### 3.1. Nurse specialist-led integrated healthcare management model

Establishment of a multidisciplinary collaboration platform and implementation of a team case discussion process. Based on the network platform to establish a group discussion WeChat group, team members in the group to contact and communicate, feedback on the nursing process of the problem, the development of personalized care process. Assessment of the child’s specialty such as nutritional status assessment, pain scores, respiratory function, etc, according to the final discussion of the treatment plan to organize a specialized nursing team to discuss the key points of care, difficulties, to the child to develop a detailed and clear care plan, the nurse in charge of the implementation of the care plan organization and fill out the MDT case discussion nursing implementation of the record sheet. During the implementation of the professional nursing problems involved in the process of each specialist nurse guidance and participation, the NICU nurse leader is responsible for the overall coordination of the work and problem summarization, in order to ensure the implementation of the team coherent care model and the improvement of emergency response capacity.

### 3.2. Personalized data management and health promotion

In order to ensure that the information between the child’s family and the MDT members is adequate and equal throughout the entire course of the disease, the nurse in charge needs to collect and organize information from both sides. During the MDT meeting, after the doctor introduces the general information about the disease, the nurse needs to provide detailed explanations, and if the doctor omits information about the patient’s medical history, concurrent comorbidities, preoperative conditions, and postoperative recovery, the nurse needs to supplement the information. If the child’s family puts forward their personal wishes (e.g., willingness to be treated, strong willingness to accept surgery, financial burden, etc) beforehand, the nurse needs to put them forward in the meeting, hoping that the doctor will consider these factors; after the meeting, the nurse needs to assist the doctor in conveying the results of the meeting to the child’s family; explaining and communicating to them the treatment plan, the process of treatment, and auxiliary treatment-related matters; and helping them to accept the treatment plan step-by-step. Due to the young age of the child and the risks associated with the surgery, families are prone to fear before surgery, so both team members should be familiar with the basic situation of the child, the type of disease, the psychological status of the family, the economic ability and support, etc, and parents’ contact, communication, use of mobile tablets, cell phone WeChat push, education albums and other forms of health education means of health education to do a good job to enhance the confidence of the family of the child’s surgical treatment. The family’s confidence in the surgical treatment was enhanced. After personalized health guidance, the families of the 4 children were informed about the disease, its treatment and care, and were able to support and cooperate with the child's treatment.

### 3.3. Goal-directed perioperative intensive care

#### 3.3.1. Key points of preoperative medication.

Maintaining the catheter open is necessary for the survival of the children; due to the introduction of prostaglandin, the survival rate of children with CHD has been improved.^[5,6]^ Prostaglandin can maintain a certain degree of blood flow in the pulmonary circulation to improve hypoxemia and effectively prevent arterial catheter closure and maintain the stability of the internal environment, with the general starting dose of 0.05 to 0.1 μg/(kg-min). Infused by constant rate infusion micropump and adjusted to the minimum effective dose after effective.^[7]^ Side effects include fever, hypotension, facial flushing, and platelet inhibition, with apnea and bradycardia being the most serious complications. The 4 children were opened to the arterial catheter with prostaglandin E1 as soon as the diagnosis was clear and the SpO2 was maintained at a stable level of 70% to 80%. Keeping away from light, the drug was pumped in a single intravenous continuous infusion, dispensed now, without interrupting the administration.^[8]^ One case in this group had hypothermia of 37.8°C to 38°C. They loosened the wrapping, lowered the temperature of the heat preservation bed, and measured the body temperature every 2 hours. The body temperature returned to normal in 1 to 2 days.

#### 3.3.2. Preoperative oxygen essentials.

Oxygen was administered cautiously to children with catheter-dependent preoperative heart disease to avoid the death of the children due to the closure of the patent ductus arteriosus caused by oxygenation. Transcutaneous oxygen saturation (SpO2) of 60% to 82% was measured preoperatively in 4 children in our group, only by the arterial catheter opening to maintain pulmonary circulation of blood, at this time, if you give a high concentration of oxygen inhalation, it can cause the arterial duct to constrict and close, leading to death, so oxygen therapy should be used with caution in nursing care. For children with preoperative respiratory distress who required ventilator-assisted ventilation, maintaining oxygen saturation at 80% was sufficient. Two children were ventilator-assisted because their SpO2 repeatedly fell below 80% after continuous pumping of prostaglandin E. Two children did not receive oxygen preoperatively.

#### 3.3.3. Catheterization room and staff preparation.

Catheterization Intervention Nurses and Roving Nurses with strong professional skills, experienced in resuscitation, and familiar with cardiovascular interventional procedures. 1 cardiovascular internal medicine nurse leader on that day focuses on this operating room, and communicates and assists in the work at any time. 2 chief surgeons of cardiovascular medicine, 1 anesthesiologist and 1 anesthesiologist each; 1 expert each from cardiac ultrasound room and radiological intervention department; 1 resident of neonatology department on standby, 1 resident of cardiovascular medicine on standby; catheterization room nurses to fully check the instruments to ensure that they can be used properly, prepare tracheal intubation, simple respirator, sputum suction and other an aesthetic accidental resuscitation supplies; temporary pacemakers, defibrillators, antiarrhythmic drugs and other resuscitation supplies. Strictly implement the measures of sterilization and isolation of goods, and prepare the pressure monitoring system, puncture supplies and surgical supplies such as arterial sheaths, catheters, lung valve balloons, and so on; Newborn babies’ body temperature center is not well developed, thin skin, blood vessel distribution, easy to dissipate heat, easy to be affected by the environment, resulting in body temperature does not rise. In the low temperature state is easy to cause circulation, metabolism and a series of changes, aggravate acidosis, coupled with severe cardiac malformation caused by hypoxemia, cardiac insufficiency, at this time to perform cardiac catheterization is prone to cause serious complications, increase the mortality rate of cardiac catheterization.^[9]^ Therefore, 1 to 2 hours before the preoperative period to do a good job of adequate thermal insulation measures, raise the temperature of the catheterization room to 24 to 26 ºC, the use of warming device 2 hours in advance to preheat, contact with the child’s mattress, cover, clothes, nappies, etc should be preheated, control the temperature of 32 to 35 ºC, humidity of 40% to 60%. Reduce the opportunity to cause a drop in body temperature.

#### 3.3.4. Active prevention of intraoperative hypothermia.

Intraoperatively, due to the temperature of the operating room, operating time, an esthesia medication, exposure of body cavities, and intraoperative irrigation. Hypothermia can lead to many adverse effects, including coagulation dysfunction, wound infection, and in severe cases, cardiac function and body metabolism.^[10]^ Therefore, comprehensive management of body temperature is needed. Specific measures: the operating room temperature is set to 25°C, and the children are covered after entering the room, and the under-padded warm air blanket is connected to the heater to keep warm at 38 to 40 ºC; after fixing the position, the warming device and preheated disinfectant towel are used to keep warm and reduce the exposure of the skin, so as to prevent intraoperative hypothermia; 4 newborns were confirmed to be in need of emergency surgery after preoperative examination, and they were fasted and given complete parenteral nutrition. Temperature was monitored at any time and recorded. None of the 4 neonates had intraoperative hypothermia, and the body temperature was maintained at 36.5 to 37.1 ºC.

#### 3.3.5. Nursing care during balloon catheter dilatation.

Intraoperative blockage of the right ventricular outflow tract by the filling balloon leads to temporary ischemia and hypoxia of the pulmonary artery, which can induce preterm systole, bradycardia, ventricular fibrillation, and even cardiac arrest.^[11]^ The performance of bradycardia, decreased oxygen saturation, decreased blood pressure, first-degree atrioventricular block, sinus arrest, ST-T changes, intraoperative catheter nurses should closely observe the child’s face, lips, nail bed color, dynamic monitoring of changes in vital signs; bedside preparation of oxygen devices, suction devices, first aid medications and defibrillators; closely observe the surgical process, timely provision of intraoperative surgical equipment and medications; in the expansion of balloon, the nurse should closely monitor and read out the balloon. During balloon expansion, the nurse should closely monitor and read out the blood pressure data of the children, observe the changes in electrocardiogram, and if the electrocardiogram is abnormal, inform the operator in time and cooperate with the first aid. They should also record the pulmonary artery pressure before and after the expansion and right ventricular pressure and the difference in the transmural pressure in detail, and save and print the pressure graph, which can be used as the basis for the evaluation of the effect of the balloon after the expansion. In 1 case of this group, heart rate and oxygen saturation decreased rapidly during intraoperative balloon dilatation, and heart rate and oxygen saturation gradually returned to normal after the administration of prostaglandin.

#### 3.3.6. Sedation, analgesic management, and developmental support care.

Early management of sedation and analgesia can effectively prevent blood pressure fluctuation and increase in myocardial oxygen consumption due to agitation in children, while avoiding postoperative bleeding.^[12]^ Four cases of children were postoperative pain assessment using the neonatal postoperative pain assessment scale (crying, mquires 02 saturation, increased vital signs, expression, and sleeplessness), postoperative assessment every 4 within 48 hours, given analgesic measures 30 to 60 minutes after the assessment again, according to the results of the assessment of the interventions given to the 4 cases of children were given postoperative midazolam 1.5 μg/(kg-min) continuous intravenous pumping; developmental supportive care, the bird’s nest imitation of the uterine environment, the use of eye masks to protect the eyes of the children, to avoid exposing the children to bright light exposure, to control the environmental noise in the neonatal ward the NICU installed sound monitors to prompt the staff in the room, to reduce the stimulation of acoustic and optical stimulation, to create a comfortable environment; Responsible nurses to record the whole process of pain management timely, accurately, objectively, and completely, including the occurrence of pain, the development of the pain, the treatment and the alleviation of the Dynamic change process. After careful and meticulous sedation and analgesia management, the 4 children had no related complications after surgery.

#### 3.3.7. Active prevention of ventilator-associated pneumonia.

Due to the characteristics of the neonatal respiratory system, the respiratory system support and management are also unique. It is very important to do a good job of ventilator-related care: should be used as much as possible for infants and young children special ventilator, the adjustment of the parameters of the ventilator should be adjusted at any time according to the results of the blood gases; in order to reduce the fluctuation of the hemodynamics of the body circulation, can be used synchronous intermittent command ventilation; to keep the tracheal intubation in the correct position, to keep the airway open, the back of the neck with a soft pillow; to strengthen the care of the airway. The correct technique of endotracheal suctioning should be mastered; the suctioning action should be gentle, the time should not be too long, and the pressure should be maintained at 0.01 to 0.02 kPa; follow the principle of asepsis when suctioning endotracheal sputum, and observe closely the changes in heart rate, blood pressure, color, and SpO2 of the children, and if there is any abnormality, stop suctioning at once and give the balloon pressurized oxygen until the SpO2 is back to normal. The children in this group were extubated from 1 to 5 days after surgery, and no ventilator-associated pneumonia occurred. In one case, hypoxemia appeared in the early stage after extubation, the oxygen saturation was about 80%, and low-flow oxygen was continuously administered, and the oxygen saturation gradually increased to 90% 3 days later, until the oxygen was completely stopped.

### 3.4. Observation and care of postoperative complications

#### 3.4.1. Pericardial effusion.

Due to the young age of the children, small blood vessels, thin wall of the blood vessels, easy to be damaged during the operation, generally choose the catheter wire as thin and soft as possible during the operation, avoiding the thicker wire in the blood vessels repeatedly probing, and strictly controlling the position of the catheter as well as the speed and pressure of the contrast. Postoperative echocardiography was routinely performed, and all children were continuously monitored for changes in heart rate, blood pressure, oxygen saturation, consciousness, pupil, and color, especially changes in heart rate and blood pressure. If the children develop symptoms such as increased heart rate, cold body, cyanosis, shortness of breath, anguished external jugular veins, and decreased blood pressure, they should be alerted to pericardial effusion, and cooperate with the doctor to perform pericardiocentesis to drain the pericardial fluid if necessary. No pericardial effusion was observed in the 4 children in this group.

#### 3.4.2. Bleeding and hematoma at the puncture site.

Bleeding at the puncture site may be caused by the relatively thin blood vessels of neonates, multiple punctures of blood vessels, intraoperative use of heparin, long operation time, damage to blood vessels by insertion of instruments, inappropriate position of pressure after extubation, insufficient time of pressure, or excessive activity of the lower limb, and so on. After the operation, we need to pay close attention to the changes in the puncture site and the limb: puncture side limb restraint brake 12 hours, with 0.5 kg sand belt pressure puncture site, and observe the local bleeding and hematoma, with gauze, elastic bandage pressure hemostasis, bandage should not be too tight, in order to touch the dorsalis pedis arterial pulsation as the standard, to avoid contamination of the dressing with urine and feces, blood seepage, timely replacement of dressings; such as the puncture site bleeding again, you should immediately use the finger pressure, and recalculate the time to press 2 hours. The temperature, color of the nail bed, and whether the dorsalis pedis artery is strong and symmetrical should be observed every 30 minutes for 2 hours after the operation and compared with the contralateral side, and then changed to every 2 hours. In this group, no bleeding occurred in the 4 cases after surgery, but all of them had different degrees of swelling of lower limbs, accompanied by skin bruising and purple coloration and lower skin temperature. Considering that the diameter of 6 F arterial sheath was thicker than femoral vein of newborn babies, which caused femoral vein injury, resulting in poor venous return to the lower limbs, we gave an elevation to the lower limbs by 15° to 30°. In the 4 cases, the swelling of lower limbs disappeared and the skin color returned to normal 1 to 3 days after the operation. On the second postoperative day, one child presented with bruising of the skin of the lower extremity on the side of the puncture, the skin temperature was increased, the fluctuation was weakened, and the ultrasound showed that there was appendage thrombosis, so the limb was braked, elevated, and subcutaneously injected with low molecular heparin calcium and other symptomatic treatments were given, and the vascular ultrasound was normal on review 1 week later.

## 4. Conclusion

PBPV is a very challenging and complex treatment, involving the knowledge of various specialties, multidisciplinary joint participation in the discussion, so that every step in the diagnosis and treatment of the child is more professional and rigorous, the nurse can not only understand the knowledge of the specialty, but also be proficient in the new theories and new technologies of related disciplines, and have a more in-depth definition of the process, systematic and comprehensive nursing.^[13]^ The implementation of the multidisciplinary collaboration model led by specialized nursing, from the implementation of the operation of the child, the precise implementation of the medical and nursing program, the postoperative enhancement of the observation and care of complications, which is conducive to the early recovery of the child, and all efforts to ensure the perioperative safety of the child, significantly improve the professional competence of nurses of all specialties, promote the development of multidisciplinary, and provide high-quality services for the children, is to improve the success rate of PBPV in neonates The key to improve the success rate of neonatal PBPV.

## Author contributions

**Writing – original draft:** Qiong He.

**Writing – review & editing:** Ling Wan, Yanping Huang, Min Song.
